# The TRIM-cancer paradox: BCG as a programmable vaccine platform and a mechanistic probe for rational immunotherapy design

**DOI:** 10.3389/fimmu.2026.1930908

**Published:** 2026-08-18

**Authors:** Victor V. Pleshkan, Liya G. Kondratyeva, Marina V. Zinovyeva, Olga A. Bezborodova, Irina V. Alekseenko, Peter V. Shegay, Andrey D. Kaprin

**Affiliations:** 1 Department of Genomics and Postgenomic Technologies, Shemyakin-Ovchinnikov Institute of Bioorganic Chemistry of the Russian Academy of Sciences, Moscow, Russia; 2 KC NBICS-Nature-Like Technologies, NRC “Kurchatov Institute”, Moscow, Russia; 3 Department of Pharmacology of Biomedical Products, P. Hertsen Moscow Oncology Research Institute, National Medical Research Radiological Centre of the Ministry of Health of the Russian Federation, Moscow, Russia; 4 Department of Oncology, Peoples’ Friendship University of Russia named after Patrice Lumumba, Moscow, Russia

**Keywords:** bacillus Calmette-Guérin, BCG, cancer, heterologous protection, immunotherapy, recombinant BCG, trained immunity, vaccines

## Abstract

BCG, a first-generation live vaccine, is being reconsidered as an immunological platform. Interest in its heterologous protection intensified during the pandemic. However, large-scale clinical trials revealed inconsistencies in the efficacy of native BCG. This review argues that BCG’s main value lies in its potential as a modifiable vector platform and in its ability to reveal tractable molecular pathways for therapeutic design. This review summarizes the molecular basis of BCG-induced trained immunity (TRIM), focusing on PRR-driven signaling, metabolic rewiring, and epigenetic remodeling in innate immune cells and hematopoietic progenitors. It also maps their convergence with pathways that sustain pro-tumorigenic inflammation. The original conceptual paradigm of the “TRIM-Cancer Paradox” is presented. This paradigm posits that the same innate immune circuits that mediate protective heterologous responses can drive tumor-promoting inflammation and immune escape under conditions of chronic dysregulation. Recombinant BCG (rBCG) is further analyzed as a strategy to rationally amplify or redirect these circuits, the current clinical landscape of BCG-based interventions across various diseases and oncological malignancies is highlighted, and specific molecular nodes that could be exploited to increase the precision, efficacy, and safety of rBCG-based therapies are identified. Overall, this review proposes BCG a programmable immunological platform and to use the TRIM–Cancer Paradox as a novel design principle for next-generation rBCG platforms that transcend traditional vaccinology and cancer immunotherapy applications.

## 1 Introduction

Bacillus Calmette-Guérin (BCG) is a live attenuated *Mycobacterium bovis* vaccine introduced in 1921 and it remains the only licensed vaccine against tuberculosis (TB), despite highly variable efficacy (1). BCG is still widely used, particularly in childhood immunization programs, and its long clinical history has made it one of the best-characterized live bacterial vaccines.

Extensive work has shown that, beyond preventing tuberculosis, BCG can provide heterologous protection against unrelated pathogens. Clinical observations suggest that neonatal BCG vaccination reduces infant mortality from all causes beyond its effect on TB (2, 3). It is thought that these non-specific benefits are mediated, at least in part, by trained immunity (TRIM), a process whereby innate immune cells become more responsive through epigenetic and metabolic reprogramming (4).

The COVID-19 pandemic provided the most rigorous real-world test of this concept. Although early ecological studies generated enthusiasm, subsequent randomized trials showed that native BCG alone does not provide reliable protection against SARS-CoV-2 infection or severe COVID-19 (5–7). This outcome should not be interpreted as a flaw in the concept of heterologous protection. Rather, it suggests that native BCG is a useful but imperfect clinical tool. By understanding the molecular mechanisms of trained immunity, we can improve BCG rationally, transforming it from a proof of concept into a precise, engineered platform for future applications.

Accordingly, this review is structured around a translational logic. Firstly, the conceptual basis for pan-pandemic interventions is considered in terms of BCG-mediated heterologous protection. Secondly, the molecular mechanisms of trained immunity are examined as potential targets for the rational reinforcement of BCG through recombinant BCG (rBCG). Thirdly, these pathways are also examined in oncology, where their acute activation can be therapeutic, but chronic dysregulation can promote tumor growth. This illustrates the TRIM-cancer paradox and the broader principle that shared innate circuits underlie protective and pathological inflammation in cancer, infections, and other immune-mediated disorders. The current clinical landscape of BCG-based interventions is analyzed to highlight the broad applicability of rBCG. To our knowledge, no previous review has systematically integrated the molecular circuitry of BCG-induced TRIM with chronic inflammatory programs that sustain tumor progression. Likewise, no review has formalized this relationship as a distinct conceptual entity. Finally, the molecular and cellular foundations of BCG action are examined to identify specific molecules and pathways that could establish BCG as a programmable immunological platform. These findings aim to articulate the “TRIM–Cancer Paradox” as a guideline for designing next-generation rBCG-based interventions.

## 2 Molecular and cellular basis of BCG-induced trained immunity

### 2.1 Molecular mechanisms of BCG-induced trained immunity and factors leading to its impairment

**Figure 1:**
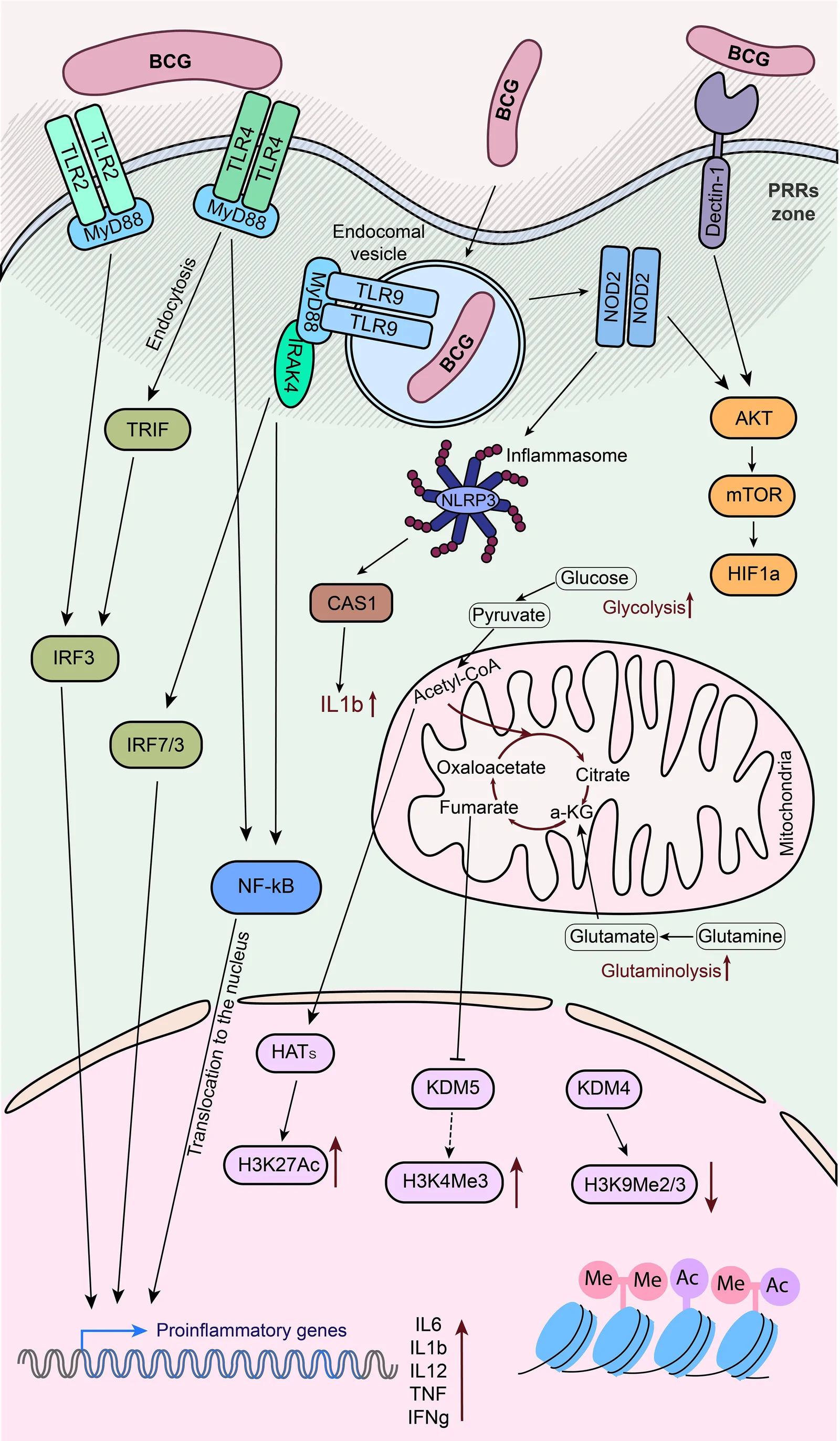
Simplified scheme of BCG-induced trained immunity. BCG engages surface (TLR2, TLR4, Dectin-1), endosomal (TLR9), and cytosolic (NOD2) pattern recognition receptors, triggering three interconnected pathways: (1) Transcriptional activation via NF-κB and IRF translocation, inducing inflammatory cytokines (IL-6, IL-1β, TNF, IL-12) and interferons; (2) Metabolic reprogramming through AKT/mTOR/HIF-1α, enhancing glycolysis and TCA cycle activity, thereby increasing acetyl-CoA and other immunometabolites; (3) Epigenetic remodeling via HATs and KDMs, depositing activating marks (H3K27ac, H3K4me3) and removing repressive marks (H3K9me3) at pro-inflammatory gene promoters. These changes establish a sustained state of enhanced responsiveness to secondary stimuli. Abbreviations: PRRs—pattern recognition receptors; TLR—Toll-like receptor; NOD2—nucleotide-binding oligomerization domain-containing protein 2; MyD88—myeloid differentiation primary response protein 88; TRIF—TIR-domain-containing adapter-inducing interferon-β; NF-κB—nuclear factor kappa B; IRF—interferon regulatory factor; mTOR—mammalian target of rapamycin; HIF-1α—hypoxia-inducible factor 1-alpha; TCA—tricarboxylic acid; Ac-CoA—acetyl coenzyme A; HATs—histone acetyltransferases; KDMs—lysine demethylases. Adapted from (19), available under a CC BY 4.0 license.

The main pathway of BCG activation is shown in **Figure 1**. BCG engagement involves immune cell pattern recognition receptors (PRRs) interacting with pathogen-associated molecular patterns (PAMPs). This triggers three interconnected processes: (1) transcriptional activation of inflammatory mediators; (2) metabolic rewiring that supports epigenetic remodeling; and (3) stable epigenetic reprogramming that primes cells for enhanced future responses.

Surface TLR2 (recognizing glycolipids and peptidoglycans), TLR4 (detecting bacterial lipopolysaccharides in heterologous settings and mycobacterial heat shock proteins), the non-TLR receptor Dectin-1 (sensing β-glucans), endosomal TLR9 (identifying unmethylated CpG DNA), and the cytosolic sensor NOD2 (sensing muramyl dipeptide) initiate signaling cascades leading to the translocation of transcription factors (NF-κB, IRFs) into the nucleus, activating the synthesis of inflammatory cytokines (IL-6, IL-1β, TNF, IL-12) and interferons (8–10). These transcriptional events provide the immediate inflammatory response required for downstream immune activation.

Simultaneously, metabolic reprogramming occurs through NOD2- and Dectin-1-dependent activation of the AKT/mTOR pathway (8–10), enhancing glycolysis (11). This glycolytic shift increases the flux of immunometabolites, including acetyl-CoA, fumarate, succinate, NAD+, and mevalonate, which function as substrates and cofactors for chromatin-modifying enzymes (12, 13). Thus, metabolism is not merely supportive but mechanistically instructive for trained immunity.

These changes culminate in epigenetic reprogramming through histone-modifying enzymes, including HATs and KDMs, leading to the accumulation of activating marks such as H3K27ac and H3K4me3 and the removal of repressive marks such as H3K9me3 at the promoters of inflammatory genes (IL6, TNF, IL-1β). This establishes an enhanced preparedness state that persists after the primary stimulus is cleared (14). The main effectors of BCG-induced TRIM and their pleiotropic effects on immune cells are summarized in **Supplementary Table 1**.

In addition, we constructed a curated cross-disciplinary matrix (**Table 1**) that systematically juxtaposes molecular factors known to modulate TRIM induction with their documented roles in cancer biology, thereby linking innate immune training to tumor-promoting or tumor-suppressive functions at the level of shared metabolic and epigenetic nodes. Factors that impair TRIM generally target these same pathways. NOD2 deficiency prevents TRIM induction after BCG exposure (15, 16). Pharmacological inhibition of mTOR by rapamycin or metformin (via AMPK activation), and of glycolysis by 2-deoxyglucose, suppresses the metabolic reprogramming required for epigenetic remodeling (17). Butyrate acts as an HDAC inhibitor and interferes with the accumulation of activating histone marks at inflammatory loci (18). These observations are important because they identify molecular nodes that may later be exploited for therapeutic reinforcement – or, conversely, may limit the efficacy of BCG-based interventions.

**Table 1:** Molecular modulators that hinder the induction of BCG-induced trained immunity (TRIM) and their context-dependent roles in cancer.

Factor	In immunity	In cancer
NOD2-deficiency	Failure of infection resistance; failure of TRIM formation (19).	Pro‐tumorigenic macrophages, promote lung adenocarcinoma progression (19).
Butyrate	Histone deacetylase 3 (HDAC3) inhibition, HDAC3 deacetylates H3K27ac and H3K9ac – active promoter/open chromatin marks (18).	Therapeutic target in cancer (20).
Fumarate	Inhibitory effect on the activity of KDM5; induces enrichment of H3K4me3 on promoters (21).	Fumarate hydratase loss is possibly pro-tumorigenic (21). Fumarate accumulation and cause hereditary leiomyomatosis and renal cell cancer, supporting its oncometabolite role (22, 23).
Ascorbate	Antioxidative and Anti-Inflammatory (24). Abrogate TRIM by inhibiting HIF1a (25).	Its antitumor role remains controversial. Although multiple studies describe anticancer activity, the evidence base remains insufficient, and as of March 2026 neither the FDA nor CDER had approved intravenous vitamin C as a cancer treatment (26–28). Facilitates tumor cell death through the generation of ROS and ferroptosis (29). Potential anti-tumor capabilities (30). Boosts DNA damage and cell death in melanoma cells (31). High concentrations triggering cell death and low amounts promoting tumor growth (32).
Rapamycin, 2-deoxyglucose (2-DG) or metformin	Inhibition of the mTOR pathway or glycolysis (17).	Rapamycin anti-cancer role discussion (33, 34). Metformin and 2-DG exhibit multiple metabolic and immunomodulatory anti-cancer effects, suppressed proliferation or PD-L1 expression (35).

Designations: NOD2 - nucleotide-binding oligomerization domain containing 2; HDAC3 - histone deacetylase 3, KDM5 – histone lysine demethylase 5, HIF1a - hypoxia-inducible factor 1-alpha, mTOR - mammalian target of rapamycin.

The metabolic intermediates that drive TRIM induction in myeloid cells converge mechanistically with effectors of tumor metabolic reprogramming (36). In the tumor microenvironment (TME), α-ketoglutarate produced from glutamine catabolism binds OXGR1 on macrophages, suppressing MHC-II antigen presentation and promoting immune evasion, while simultaneously driving JMJD3-dependent epigenetic reprogramming that sustains the M2 pro-tumorigenic phenotype (37). Glutamine synthetase (GS) inhibition in tumor-associated macrophages (TAMs) shifts this metabolic balance by reducing intracellular glutamine, elevating succinate, and activating HIF-1α — thereby repolarizing M2 TAMs toward an M1-like state, normalizing tumor vasculature, and suppressing metastasis (38). Cancer cell-derived succinate further reinforces immunosuppression through SUCNR1-mediated PI3K–HIF-1α signaling in TAMs (39), precisely mirroring the succinate–HIF-1α axis that mediates IL-1β-driven TRIM in BCG-activated macrophages (13). Fumarate, which enriches H3K4me3 at pro-inflammatory gene promoters via KDM5 inhibition in TRIM (21), operates as an oncometabolite when fumarate hydratase is lost (22). This mechanistic convergence underscores that rBCG-based amplification of TRIM must achieve cell-context specificity to avoid inadvertently conditioning the TME toward immunosuppression.

### 2.2 Cells of the immune system in BCG-induced TRIM

Trained immunity manifests across multiple levels of the immune hierarchy, from hematopoietic progenitors to mature peripheral effector cells. In mice, BCG vaccination biases hematopoietic stem cells toward myelopoiesis through IFNγ-dependent mechanisms, generating trained differentiated progeny and increasing chromatin accessibility in bone marrow progenitors (40). Comparable effects have been documented in humans, where transcriptional changes in hematopoietic stem and progenitor cells (HSPCs) remain detectable months after vaccination (41).

Peripheral trained immunity is observed in tissue-resident macrophages and circulating innate immune cells. For example, alveolar macrophages from BCG-vaccinated mice display a trained phenotype and enhanced responses to heterologous stimuli that correlate with stable metabolic and epigenetic reprogramming (42). In healthy volunteers, PBMCs obtained after BCG vaccination produce increased TNF, IL-6, and IL-1β upon stimulation with *M. tuberculosis (Mtb)* lysate and unrelated pathogens, and this heightened cytokine capacity correlates with increased H3K4me3 at inflammatory loci in circulating monocytes (43).

BCG vaccination also affects NK cells and γδ T cells. NK cells acquire enhanced pro-inflammatory responsiveness to heterologous stimuli, and specific KLRG1^+^ subsets display memory-like IFNγ responses upon restimulation with mycobacterial and HIV antigens (44). γδ T cells show altered transcriptional states and enhanced TNF and IFNγ production after vaccination, placing them at the interface between innate training and adaptive amplification (45).

Optimal TRIM efficacy requires crosstalk between innate and adaptive immunity. Antigen-presenting cells (APCs) activated by BCG present antigens on MHC molecules, engage costimulatory pairs (CD80/CD86, CD40), and produce cytokines while sensing metabolic alterations (46–48). BCG vaccination enhances APC activation, leading to improved T- and B-cell engagement and increased cytokine production. Metabolic changes induced by BCG, such as increased glycolysis, rapid ATP production, and elevated reactive oxygen species (ROS) production, support the proliferation and pro-inflammatory functions observed in both innate and adaptive participants of the interaction (12, 46, 49, 50). This interplay is particularly important because interventions that strengthen trained immunity can also reshape antigen-specific immunity.

## 3 Heterologous protection and the BCG platform for pan-pandemic vaccines

### 3.1 The pan-pandemic vaccine concept

The unpredictability of future outbreaks creates a persistent gap between the emergence of a novel pathogen and the development of a pathogen-specific vaccine. This gap gives rise to the concept of a temporary protective intervention based on non-specific immune activation.

In this context, “pan-pandemic vaccines” are defined as interventions that induce trained immunity, providing temporary protection against initially unknown pathogens until pathogen-specific vaccines become available. This term is more precise than “universal vaccine” in the present context and helps distinguish emergency prophylaxis from anticancer vaccination.

BCG is the logical starting point for this concept because it is the best-studied inducer of trained immunity in clinical settings. However, available data suggest that it should be considered a proof-of-concept platform rather than a definitive pan-pandemic solution.

### 3.2 The role of BCG in modern tuberculosis vaccination and the need for improvement

The WHO recommends administering BCG at birth in regions with a high incidence of TB and/or leprosy. Although the response magnitude varies across populations and geography, birth vaccination induces stronger CD4^+^ and CD8^+^ T-cell responses than delayed administration (51). BCG is effective in protecting infants against severe TB, but its efficacy decreases in adolescents and adults, necessitating booster or replacement strategies (2).

Alternative delivery routes have been explored to improve safety, compliance, and mucosal immunity. Oral and mucosal formulations may offer practical advantages, but they face challenges including gastric degradation, antigen instability, and poor mucus penetration (43, 52–54). Numerous next-generation TB vaccine candidates have entered development, yet none has displaced BCG in clinical practice (55). This persistent failure supports a parallel strategy focused on improving BCG itself.

Thus, major efforts focus on revaccination strategies and next-generation BCG derivatives. The recombinant strain rBCGΔureC::hly (VPM1002) represents one of the most advanced platforms in this category. In conventional BCG, the urease C enzyme maintains pH stability within the host cell phagosome, preventing its acidification. In this engineered strain, the disruption of the urease C gene allows the phagosome to acidify normally, dropping the pH to approximately 5.5. This acidic environment activates the integrated listeriolysin O (LLO) protein, a cholesterol-dependent cytolysin derived from *Listeria monocytogenes*. Activated LLO perforates the phagosomal membrane, facilitating the translocation of mycobacterial antigens directly into the host cell cytosol. Consequently, these antigens are efficiently routed to the MHC class I pathway in addition to the standard MHC class II pathway, leading to a significantly enhanced induction of both CD4^+^ and CD8^+^ T-cell-mediated immune responses (56). As a result of this enhanced immunogenicity, VPM1002 has progressed through multiple clinical studies, including Phase III programs (57, 58). Its fate remains informative for the field because it tests whether rational engineering can convert BCG from a historically useful vaccine into a superior modern platform.

### 3.3 Lessons from COVID-19 and heterologous protection against other pathogens

#### 3.3.1 Heterologous protection induced by BCG

Trained immunity provides cross-protection against various pathogens after stimulation by bacteria, fungi, or viruses, thereby protecting against unrelated infections (12, 14). The BCG vaccine underscores the role of innate immune cells in protecting against acute respiratory infections in individuals of all ages. In Guinea-Bissau, measles-naïve infants with acute lower respiratory infections were more likely to be BCG-negative and lack a BCG scar (59). Among elderly individuals (60–75 years old), BCG significantly reduced acute upper respiratory infections and increased IFNγ and IL-10 levels compared with the placebo group (60). Subcutaneous BCG enhances innate protection against *Streptococcus pneumoniae* by increasing pulmonary neutrophils independently of circulating monocytes, providing insight into strategies against bacterial respiratory pathogens (61). Certain BCG strains lower overall mortality by protecting against various pathogens, including herpes simplex virus and influenza virus, in mouse models (62).

In a controlled malaria infection study, BCG-vaccinated individuals exhibited earlier and more severe clinical manifestations, as well as accelerated NK-cell and monocyte activation (63). BCG also protects against the attenuated yellow fever virus. Reduced viremia correlates with increased IL-1β, a key mediator of TRIM and epigenetic reprogramming in monocytes (64).

These findings support the view that BCG can provide a temporary state of heightened immune preparedness. However, the breadth of heterologous protection should not be confused with uniform clinical reliability.

#### 3.3.2 COVID-19 as a stress test for heterologic protection

Early ecological analyses suggested that countries with universal BCG vaccination policies experienced lower COVID-19 mortality (65, 66). However, these studies were inherently confounded by numerous socioeconomic and healthcare-system variables, and causality could not be inferred (67). This hypothesis prompted more than a dozen randomized controlled trials (RCTs) globally (12).

The results of these RCTs largely failed to confirm the promise of the early data. Two of the largest and most rigorously designed trials, the BRACE trial in healthcare workers (NCT04327206) and a similar trial conducted in the Netherlands (NCT04328441), found no significant difference between the BCG and placebo groups in the incidence of COVID-19, rates of hospitalization, or disease severity (68, 69). A study in South Africa corroborated these findings, showing no protective effect against SARS-CoV-2 infection in healthcare workers (70). These negative results contrast with a smaller Phase III trial in patients with type 1 diabetes, which reported significant protection from multi-dose BCG (71), and the ACTIVATE-2 study in older adults, which showed a relative risk reduction (72).

The discrepancy in outcomes highlights the multiple factors that likely modulate BCG’s non-specific effects, including strain (73), route and timing of administration (6, 74), host genetics and immune status, and potentially the specific pathogen in question. The key lesson is therefore not that trained immunity lacks translational value, but that native BCG is too context-dependent to serve as a robust standalone pan-pandemic intervention.

### 3.4 Reinforcement of non-specific BCG protection

The reinforcement of non-specific protection by BCG involves overlapping innate and adaptive mechanisms. BCG can trigger rapid PRR-dependent inflammatory responses and heterologous immunity, including cross-reactive T cells. Broadly, three strategies are evident: booster approaches using adjuvants, engineering of recombinant BCG strains, and combination regimens that use BCG as a priming platform.

#### 3.4.1 Booster strategy

Combining attenuated vaccines with adjuvants is a straightforward way to increase the magnitude of non-specific immune activation (75). The use of adjuvants aims to increase the amplitude of the non-specific immune response and promote a more stable induction of TRIM.

The combination of alum and BCG has been explored in vaccines against visceral leishmaniasis (VL). Pilot studies conducted in Sudan showed that first-generation vaccines for VL based on a combination of autoclaved Leishmania major (ALM) and BCG were safe, but did not exceed BCG alone in protective properties (76). Subsequent attempts to enhance this formulation by adding alum suggested only modest improvement, and no licensed human vaccine for leishmaniasis is yet available (77–79).

These results suggest that adjuvanting BCG can amplify immune activation. However, they also highlight the limited predictability of simple, amplitude-based enhancement.

#### 3.4.2 Recombinant BCG

The most rational strategy for strengthening BCG appears to be recombinant engineering. rBCG strains can be designed to encode additional cytokines, chemokines, antigens, or immune-modulating molecules (80), thereby turning BCG into a programmable immunological chassis.

The genetic architecture of recombinant BCG platforms relies on two distinct strategies: chromosomal integration and plasmid-based expression. Genomic integration, typically mediated by site-specific recombination, ensures long-term monoclonal stability; however, it often results in low-level expression. In contrast, replicative shuttle vectors leverage high copy numbers to maximize antigen yield. However, they are prone to segregation instability and plasmid loss *in vivo* due to the absence of continuous selective pressure.

One important design class consists of cytokine- or chemokine-expressing rBCG strains that improve the recruitment and differentiation of APCs and T cells. BCG/MCP-3 enhances lymphocyte migration and antigen-specific T-cell responses (81). BCG/mGM-CSF provides markedly superior protection against disseminated *Mtb* infection compared with parental BCG and increases APC accumulation and IL-12 production in draining lymph nodes, thereby improving CD4^+^ T-cell priming (82). Intranasal delivery of GM-CSF-expressing rBCG also enhances pulmonary DC responses and improves survival. These findings show that rBCG-encoded cytokines can locally amplify antigen presentation and T-cell priming while avoiding systemic inflammatory toxicity.

A second design class seeks to reinforce trained immunity directly. rBCG vaccines expressing microbial or viral proteins have been shown to induce TRIM and enhance protection (83). For instance, rBCG expressing the S1 subunit of pertussis toxin (rBCG-S1PT) provided 100% protection against an intracerebral *Bordetella pertussis* challenge in newborn mice and cross-protected against *Candida albicans* (84, 85). The combination of rBCG with viral vector boosters enhanced T-cell responses against HIV (86). rBCG expressing the hMPV phosphoprotein reduced viral load and tissue damage (87–89), while rBCG expressing the hRSV nucleoprotein conferred protection through Th1/Th17 memory and neutralizing antibodies (90).

Mucosal vaccination with rBCGPPE27 enhances glycolytic metabolism, innate cytokine production, and heterologous protection in mice, with mechanistic dependence on mTORC2 and hexokinase 1 (HK1) (42). This is particularly important because it directly links recombinant BCG engineering to the molecular core of trained immunity rather than merely to stronger antigen-specific responses.

A third strategy combines rBCG priming with conventional protein or adjuvant boosters. In transgenic mouse models, rBCG-ChD6 priming followed by rChimera-Alum boosting enhanced neutralizing antibodies, cytokine responses, and control of a SARS-CoV-2 challenge relative to controls (91, 92). Taken together, these data support three non-exclusive roles for rBCG: a strengthened BCG platform, an active TRIM amplifier, and a vector for additional immune instructions.

## 4 Introducing the TRIM-cancer paradox

### 4.1 Shared pathways, divergent outcomes

The same molecular circuitry that makes BCG attractive for anti-infective immune priming also creates a major conceptual problem in oncology (**Supplementary Table 1**). We propose the term “TRIM-Cancer Paradox” to describe the critical duality whereby the same effector pathways that mediate protective responses against pathogens and tumors can actively promote malignancy when they are chronically dysregulated within the tumor microenvironment. This phenomenon exemplifies tumor-promoting inflammation as a cancer hallmark-enabling characteristic that supports the acquisition and maintenance of multiple malignant capabilities (93).

BCG’s antitumor efficacy relies primarily on the acute, local activation of pattern recognition receptors (PRRs). This danger signal generates a potent pro-inflammatory milieu characterized by rapid cytokine release (IL-1β, TNF, IL-6, IFNs), the recruitment and activation of innate immune cells (neutrophils, macrophages, NK cells), and subsequent priming of adaptive immunity (12, 94). This immediate inflammatory response, rather than trained immunity per se, is the principal driver of tumor clearance. TRIM – the long-term epigenetic and metabolic reprogramming of innate immune cells may contribute to the durability of this antitumor effect by maintaining a heightened state of immune vigilance, but it is not the primary mechanism of tumor elimination.

However, these same mediators are frequently associated with malignant progression when their activity becomes chronic or dysregulated within the tumor microenvironment. Persistent IL-1β promotes MDSC accumulation, M2-like polarization, and metastasis (95–97). Chronic TNF signaling supports NF-κB-dependent survival, invasion, and angiogenesis (98–101). Interferons can induce PD-L1, IDO, and other adaptive resistance programs and they may support cancer stemness or therapy resistance (102–104). Thus, the challenge in BCG-based oncology is not merely to increase inflammation, but to confine it in time, space, and function.

This duality aligns with the classic conceptualization of a tumor as a “non-healing wound” – a site of persistent inflammatory signaling that fails to resolve. In this context, factors that promote TRIM induction may, under conditions of chronic stimulation, become pro-tumorigenic.

Nevertheless, the existing literature has largely left this duality untranslated into actionable design principles. Although several contemporary reviews have explored the intersection of trained immunity and oncology, none has formalized this inherent duality as a structured concept for therapeutic engineering. Some accounts present TRIM primarily as an amplifier of antitumor immunity, emphasizing synergy with checkpoint inhibitors but overlooking its pathological potential (105). Others systematically catalog its pro-tumorigenic consequences, yet stop short of proposing a unified strategy for managing signal context (106). The pathological consequences of TRIM in chronic inflammatory conditions, such as renal disease and transplantation, have been outlined, but this perspective has not been applied to the tumor microenvironment (13). Moreover, a recent systematic appraisal of BCG-induced heterologous protection underscores that clinical outcomes remain highly context-dependent, reinforcing the need for controllable interventions (107). Thus, the TRIM-Cancer Paradox, as articulated here, is not merely a restatement of the dual role of inflammation. Rather, it provides an operational principle for the rational engineering of recombinant BCG platforms. This integrative approach, which connects the molecular circuitry of innate immunity directly to rBCG design, constitutes the original contribution of this work.

Recent clinical observations underscore this ambiguity. Intravesical BCG in bladder cancer patients induces persistent heterologous responsiveness in PBMCs, consistent with trained immunity (108). Whether this sustained imprint contributes to durable tumor control or, under some conditions, could reinforce tumor-promoting inflammation remains unresolved.

### 4.2 Autophagy perplexity

Autophagy adds a second layer of mechanistic complexity. BCG can induce autophagy and apoptosis in tumor models, and the autophagic machinery has been implicated in monocyte-to-macrophage differentiation and antitumor macrophage function (109–112). Yet autophagy can also support tumor cell survival under stress in advanced malignancies (113).

This ambiguity is mirrored pharmacologically. Rapamycin promotes autophagy through mTORC1 inhibition and is frequently discussed as an anticancer agent (33, 34, 114, 115), but it also suppresses trained immunity by blocking the metabolic program required for TRIM (**Table 1**). Therefore, the enhancement of autophagy cannot be assumed to be uniformly beneficial across BCG-based applications.

BCG can also block autophagy in macrophages to promote mycobacterial persistence, while autophagy induction can counteract this effect (116, 117). Recombinant strains expressing additional PRR activators may improve autophagy and antigen presentation, and the deletion of autophagy-blocking genomic elements has been proposed as a design strategy (118, 119). Again, recombinant engineering appears to be the most reasonable approach for transforming a biologically intricate parental strain into a more controllable therapeutic tool.

## 5 Cancer immunotherapy and the clinical landscape of BCG

### 5.1 Spurring antitumor immunity

A major aim of cancer immunotherapy is to render tumors visible and vulnerable to the immune system. BCG is attractive because it functions as a non-lethal pathogen-derived danger signal that activates multiple PRRs simultaneously. This makes it well suited to disrupt local immune tolerance and initiate broader antitumor responses.

BCG became the first FDA-approved immunotherapy in 1990 and remains the standard intravesical treatment for high-risk non-muscle-invasive bladder cancer (NMIBC) (120–122). Of note, rBCG strains have entered clinical development in oncology. VPM1002BC, derived from the rBCG strain VPM1002, is being investigated for the intravesical therapy of NMIBC after recurrence following conventional BCG therapy (57, 123, 124). Compared with the conventional BCG strains, VPM1002BC promotes phagosomal escape and enhances cross-presentation to CD8^+^ T cells. This is a major translational milestone because it represents a clinically relevant engineered attempt to improve BCG through a defined molecular mechanism.

Other tumors, including melanoma, lymphoma, renal cell carcinoma, and prostate cancer, have also shown varying degrees of responsiveness in preclinical or clinical contexts (125–127). Infant BCG vaccination has been shown to reduce the risk of cancer and mortality from malignancies such as leukemia and lung cancer (128–130). The latter observation suggests that BCG vaccination may exert a protective antitumor effect, potentially related to processes similar to those involved in heterologous protection against infections (131). These data support the broader immunotherapeutic relevance of BCG, although bladder cancer remains its clearest clinical success.

### 5.2 Merging the antitumor features of BCG

The antitumor action of BCG stems from several partially overlapping mechanisms that converge. First, an acute PRR-driven inflammation creates a local “danger signal” environment that breaks immune tolerance and initiates tumor-directed immunity. Second, TRIM-associated metabolic and epigenetic rewiring may prolong immune vigilance; however, this trained immunity is not the primary driver of tumor clearance. Third, the enhancement of autophagy and antigen presentation can improve the cross-priming of CD8^+^ T cells, particularly with engineered rBCG strains.

This convergence explains why BCG can be both effective and difficult to optimize. Its utility derives from the breadth of pathways it activates; however, its limitations arise from this same breadth because some of these effector pathways can become harmful when persistent. Chronic dysregulation of IL-1β, TNF, IL-6, and IFNs within the tumor microenvironment can promote immunosuppression, angiogenesis, and malignant progression – a duality termed the “TRIM-Cancer Paradox”.

The challenge and opportunity are in designing interventions that harness BCG’s immune-activating power while controlling the inflammation it causes. This requires the precise engineering of rBCG constructs, rational combination with immune checkpoint inhibitors or anti-inflammatory modulators, and careful attention to dosing and timing. An important extension of this concept is recognizing that intravesical BCG activates not only local PRRs but also reprograms bone marrow HSPCs systemically. This central TRIM mechanism amplifies the antitumor output of myeloid cells and could expand the potential of BCG-based immunotherapy to treat micrometastatic disease.

### 5.3 HSPC reprogramming and systemic antitumor myelopoiesis: mechanistic extension and rBCG design implications

A mechanistically pivotal advance is the demonstration that intravesical BCG colonizes the bone marrow and reprograms HSPCs. This establishes a systemic antitumor circuit that extends beyond the local urothelial compartment. Using integrated PBMC profiling with transcriptomic and chromatin accessibility analyses, it was shown that intravesical BCG induces an IFNγ-dependent transcriptional program in HSPCs. This program augments myelopoiesis and biases differentiated progeny toward pro-inflammatory, antigen-presenting phenotypes. BCG was cultured directly from murine bone marrow, strongly pointing toward hematogenous dissemination as a key prerequisite for this mechanism (132). BCG-reprogrammed HSPCs produced neutrophils, monocytes, and dendritic cells (DCs) with altered functional states. Trained neutrophils derived from BCG-reprogrammed HSPCs exhibit resistance to TME-driven reprogramming toward the pro-tumorigenic T3 subset, maintaining their mature T2 non-tumorigenic identity. Trained monocytes upregulate TNF, IL-6, and CXCL10. This antitumor activity was dependent on CD8^+^ CTLs and synergized with anti-PD-1/PD-L1 checkpoint blockade in murine models. These findings support a model in which bone marrow reprogramming operates upstream of and potentiates adaptive immunity.

These findings reveal a key TRIM mechanism: HSPC-level epigenetic imprinting. This mechanism is distinct from the peripheral TRIM in tissue-resident macrophages, which was described in Section 2.2. The chronically dysregulated counterpart of this mechanism corresponds precisely to the pro-tumorigenic arm of the TRIM-Cancer Paradox.

The high response rates observed in the SAKK 06/19 trial, particularly the significant improvement compared to the BCG-free predecessor trial (SAKK 06/17), provide clinical support for the HSPC-centered trained immunity concept. These results are consistent with a systemic antitumor effect mediated by BCG-reprogrammed myeloid progenitors, rather than local action alone. In this single-arm, phase II trial, the rBCG strain VPM1002BC (rBCGΔureC::hly) was combined with three weekly intravesical instillations, atezolizumab, and gemcitabine/cisplatin in patients with muscle-invasive bladder cancer (MIBC). The primary analysis showed a pathologic complete response (pCR) rate of 68%, a pathologic overall response (PaR) rate of 83%, and 12-month event-free survival (EFS) and overall survival (OS) rates of 90% and 96%, respectively (133). The observed induction of chemoattractant cytokines and cytotoxic molecules (e.g., CXCL9, CCL19, CXCL13, perforin, granzyme B) following rBCG and atezolizumab supports the notion that rBCG elicits a systemic immune response, which may account for its antitumor activity beyond the bladder and is in line with the HSPC-centered trained immunity mechanism.

An unresolved aspect of the HSPC-centered model is the route by which intravesical BCG or BCG-laden inflammatory cells reach the bone marrow. Although colonization of the bone marrow by intravesical BCG has been explicitly demonstrated, the exact trafficking pathway remains undefined, suggesting hematogenous dissemination as the primary route (132). Independent work in melanoma has established that an intact VEGF-C/VEGFR3-dependent lymphatic network is indispensable for efficient transport of antigen and dendritic cells to draining lymph nodes and for generating a T cell–inflamed tumor microenvironment (134). Furthermore, tumor-associated lymphangiogenesis can promote recruitment of naïve T cells and potentiate checkpoint blockade (135). In parallel, lymph node LECs archive antigen through a defined gene-expression program, extending the temporal window of antigen availability for T-cell restimulation (136).

While these data do not directly implicate lymphatic routes in BCG delivery to the bone marrow, they provide a strong mechanistic precedent. Specifically, BCG-exposed or trained myeloid and T cells may exit the bladder wall via lymphatic vessels, transit through draining lymph nodes for antigen archiving, and subsequently re-enter the bloodstream to reach bone marrow niches. This model remains a hypothesis until experimentally verified. Nevertheless, it establishes the lymphatic system as a rational design target for next-generation TRIM therapies.

Consequently, reinforcing the IFNγ-dependent trained immunity program at the level of HSPCs through rationally engineered rBCG constructs that enhance cytosolic DNA sensing and inflammasome activation in myeloid precursors could strengthen cGAS–STING signaling. Such design modifications could improve the antitumor epigenetic imprint while limiting MDSC expansion and M2 TAM polarization (106).

Taken together, this interplay between central HSPC reprogramming and potential lymphatic trafficking provides a speculative yet compelling mechanistic rationale for the shifting oncological trend toward combination regimens. The integration of BCG with immune checkpoint inhibitors (ICIs) targeting PD-1/PD-L1 reflects a notable paradigm shift, supported by emerging clinical efficacy trends. Mechanistically, BCG-induced inflammation can convert an immunologically “cold” environment into a more responsive, “hot” tumor microenvironment. By promoting T-cell infiltration and upregulating checkpoint molecules, this combination may sensitize otherwise recalcitrant tumors to ICI therapy, potentially expanding the proportion of responding patients (137–139).

### 5.4 Current clinical trials with BCG

**Figure 2:**
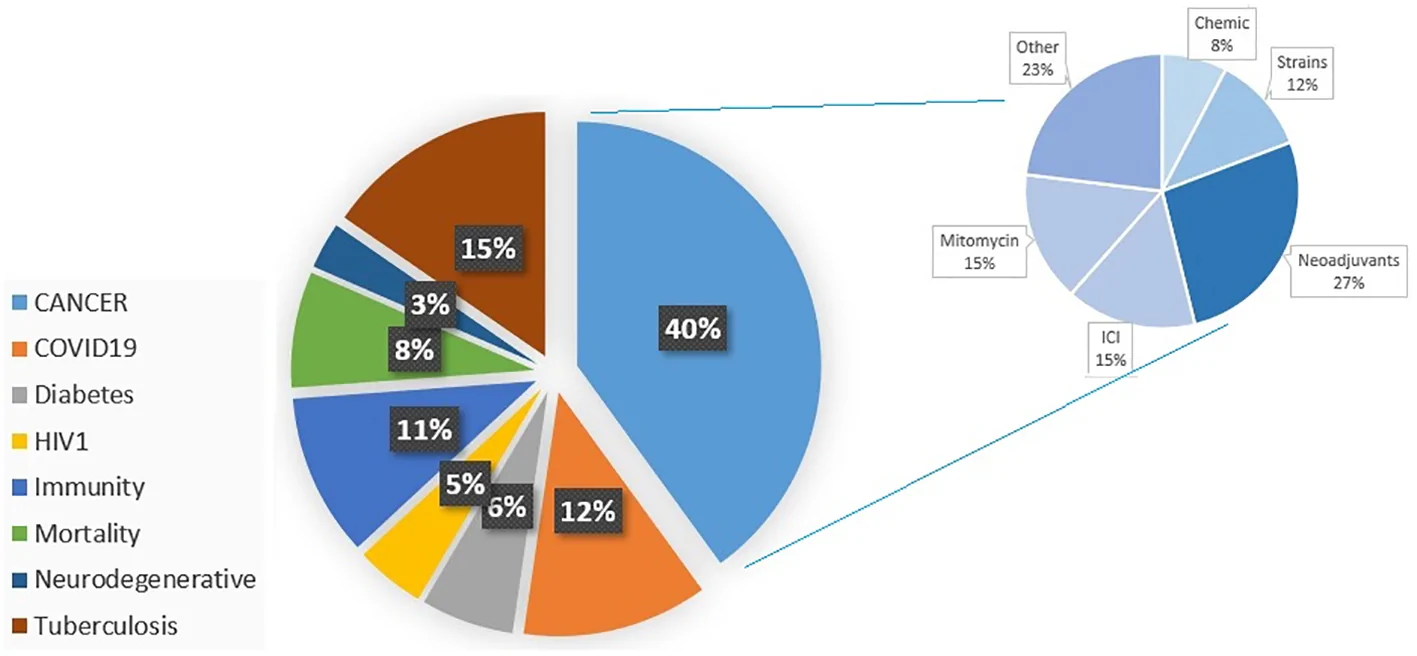
Distribution of actual clinical trials with BCG. The distribution by conditions of current clinical trials using BCG is shown. A total of 65 CTs were analyzed. The diagram on the right shows the distribution by BCG use options for the cancer group. The distribution by therapeutic area is shown, with cancer trials representing the largest proportion (n=26, 40%), followed by tuberculosis (n=10, 15%). Other indications include diabetes, HIV, immunity, mortality, neurodegenerative disorders, and COVID-19. The inset shows the distribution of most prominent BCG combination strategies within cancer trials.

Beyond the main areas of oncology, tuberculosis, and heterologous protection against COVID-19, the unique immunological properties of BCG are being actively investigated across a diverse range of clinical niches. A survey of active and recent clinical trials reveals additional applications in diabetes, HIV, heterologous immunity, mortality, and neurodegenerative diseases (**Figure 2**). This broad clinical landscape reflects the multipronged immune activation capacity of BCG. Although current trials often use native BCG, their objectives, which range from systemic immunometabolic reprogramming to neuroimmune modulation, formally align with the capabilities of rBCG platforms. These “non-canonical” applications represent promising future niches for rBCG-based interventions.

#### 5.4.1 Diabetes: metabolic repurposing

The ability of BCG to induce aerobic glycolysis has prompted research into type 1 diabetes (T1D). Repeated administration is associated with reduced blood glucose levels, the restoration of regulatory T cell gene expression, and a systemic shift toward aerobic glycolysis (140, 141). This application is noteworthy because it broadens the scope of BCG use beyond the treatment of infection and cancer. Furthermore, it aligns with BCG’s impact on glycolysis and the durable systemic immunometabolic reprogramming involved in trained immunity.

#### 5.4.2 HIV: safety and immune activation

HIV-related studies focus mainly on safety in HIV-exposed or infected infants and on the balance between beneficial non-specific immune activation and the risk of exacerbating disease progression or susceptibility to disseminated BCG infection (142).

#### 5.4.3 Heterologous immunity, mortality, and neurodegeneration

Trials on infant mortality and heterologous protection continue to evaluate whether BCG reduces all-cause mortality beyond tuberculosis prevention, but the results remain heterogeneous across settings (143–147). Retrospective observations have also linked intravesical BCG therapy with a reduced incidence of Alzheimer’s disease, raising the possibility that BCG-mediated immunoregulation may influence neuroinflammation (148–150). Proposed mechanisms include the BCG-induced elevation of IL-2 and the expansion of regulatory T cells, which may suppress neuroinflammation. These emerging fields remain provocative but preliminary.

## 6 BCG as a programmable platform: integrating trained immunity and the TRIM-cancer paradox into rational immunotherapy design

The pandemic clarified a central point of this review: native BCG is not a reliable, universal intervention for all pandemics. However, it remains the most informative proof-of-concept platform for understanding and exploiting trained immunity. Instead of asking whether BCG itself can serve as a universal solution, a more productive question is how its immunological logic can be refined, redirected, and made clinically controllable. In this regard, BCG can be considered both a modifiable vector platform and an experimental system that reveals actionable intersections between innate training, inflammation, and pathological immune remodeling.

This repositioning is important because the same BCG-triggered pathways can support distinct outcomes depending on the context, timing, and persistence of activation. In infection and emergency prophylaxis, the objective is to induce a rapid but controlled state of heightened innate responsiveness. In cancer, however, the same inflammatory mediators and metabolic circuits require interpretation through the lens of the TRIM-Cancer paradox. Pathways that contribute to acute tumor-disruptive immunity may support angiogenesis, immune escape, and tissue remodeling under chronic or poorly resolved activation. Thus, the paradox is not a contradiction of BCG efficacy, but rather a conceptual tool for distinguishing beneficial immune activation from its pathological continuation.

The primary value of rBCG extends beyond strengthening BCG itself. It provides a means to reshape immune activation by introducing defined signals into a platform that engages innate pattern-recognition networks. This creates an opportunity to move from the empirical use of BCG toward rationally designed immunobiologicals.

The logic also expands beyond BCG as a product. Once the relevant BCG-activated pathways are identified, they can serve as a blueprint for therapeutic intervention. Consequently, BCG functions as both a candidate drug platform and a biological reference model for discovering novel mechanisms of immune regulation across PRR signaling, immunometabolic rewiring, and epigenetic remodeling.

Accordingly, future BCG-based therapeutics should incorporate control over the key shared signaling pathways highlighted by the TRIM-Cancer paradox. The aim is to direct immune activation toward a favorable outcome: sufficient induction of protective innate memory and antitumor inflammation, without establishing persistent signaling states that promote immune dysfunction or pro-tumorigenic remodeling. This systems view treats the immune response as an integrated cascade initiated by a single trigger and propagated across multiple cellular compartments.

In this way, several translational directions make sense. One is engineering rBCG strains carrying cytokine or chemokine payloads that enhance APC recruitment, local innate activation, or functional coupling between innate and adaptive immunity. Another is using BCG-derived mechanistic knowledge to identify druggable nodes that extend beyond the bacterial platform. A third is a combinatorial design, in which BCG or rBCG provides the initial inflammatory and training signal, while additional components shape magnitude, tissue specificity, or resolution. These options should be selected according to the intended application rather than being treated as universally interchangeable solutions.

Thus, the central outcome of this conceptual review is the repositioning of BCG as a modern immunological platform and a mechanistic entry point into the biology of trained immunity. In this light, the TRIM-Cancer paradox is not merely an observation about cancer-related inflammation, but a guiding principle for therapeutic design. To operationalize this principle, **Supplementary Table 2** aligns TRIM-modulating targets and agents with their expected pro- or anti-tumor (and, when applicable, metabolic) effects. This provides a practical matrix for selecting candidates for rBCG genetic engineering within the TRIM-Cancer Paradox paradigm. Ultimately, the clinical value of BCG lies in its capacity to illuminate these targeted intervention opportunities across oncology and other immune-mediated diseases.

## 7 Limitations and challenges of BCG-based interventions

Although recombinant BCG (rBCG) provides a promising foundation for the development of rational immunotherapies, the transition from laboratory research to clinical application is hindered by various biological, technological, practical, and safety-related limitations. These challenges are particularly pronounced in the context of intravesical administration for bladder cancer, the most well-established clinical application of BCG immunotherapy, but they also apply to all other proposed uses of BCG as a programmable immunological platform.

### 7.1 Strain-dependent variability in efficacy and reactogenicity

The heterogenous outcomes observed in BCG-induced non-specific immune responses stem from a combination of inherent vaccine limitations, genetic strain variability, and environmental factors. BCG is not a perfect vaccine and possesses two major limitations: its efficacy against TB varies considerably between pediatric and adult populations, and it can cause disseminated BCG disease in immunocompromised individuals. Although multiple genetically distinct substrains exist, the precise clinical impact of their genomic divergence is not fully understood due to a lack of adequately powered comparative trials (151). However, a comparative analysis of 13 BCG strains in SCID and BALB/c mice demonstrated that virulence and protective efficacy are lineage-dependent; specifically, the DU2 IV group strains (BCG-Phipps, BCG-Frappier, BCG-Pasteur, BCG-Tice) exhibited the highest virulence and subsequent protection against *M. tuberculosis* challenge, whereas the DU2 II group strains (BCG-Sweden, BCG-Birkhaug) displayed the lowest (73), with these differences driven by strain-specific genomic duplications and deletions.

Beyond genetics, recent clinical data indicate that the season of vaccination significantly modulates host immunity. Winter vaccination led to a greater increase in pro-inflammatory cytokine production by PBMCs compared to spring but resulted in lower IFNγ release. NK cells from winter-vaccinated individuals showed an enhanced capacity to produce pro-inflammatory cytokines and IFNγ upon stimulation. While vaccination had minimal impact on the monocyte transcriptome at 3 months, seasonal epigenetic changes in monocytes and NK cells were observed, explaining the heightened immune reactivity in the winter group. These findings suggest that winter BCG vaccination enhances the trained immune response through the activation and reprogramming of immune cells, particularly NK cells (Dutch clinical trial registry no. NL58219.091.16) (74).

### 7.2 The miRNA barrier

Another fundamental technological constraint is the inability of BCG to directly serve as an expression vector for eukaryotic microRNA (miRNA) precursors. Although miRNAs are powerful tools for modulating host immune responses and oncogenic pathways (**Supplementary Table 2**), their biogenesis requires primary transcript processing by Drosha and Dicer enzymatic complexes, which are absent in prokaryotic systems. Consequently, no studies to date demonstrate the direct bacterial synthesis of functional miRNA precursors by BCG (80). While BCG infection naturally modulates host cellular miRNA profiles (152, 153), this shift occurs via the indirect manipulation of host signaling cascades rather than the transgenic delivery of RNA interference (RNAi) machinery. Overcoming this barrier would necessitate highly intricate engineering strategies. These approaches remain entirely speculative and face severe translational hurdles regarding processing fidelity and functional integration, thereby restricting the rational design scope of the rBCG platform.

### 7.3 Practical and logistical constraints

From a clinical logistics perspective, intravesical BCG therapy imposes significant infrastructure demands. Administration requires specialized rooms equipped for instillation procedures, stringent aseptic techniques, and rigorous protocols for the handling and disposal of live mycobacteria as biohazard material. Healthcare personnel face occupational exposure risks during preparation and administration, which necessitates comprehensive training and adherence to strict infection prevention measures. These requirements limit the widespread adoption of BCG therapy.

### 7.4 Physiological burden and safety concerns

The physiological burden on patients represents another critical constraint. Intravesical BCG is associated with a high incidence of adverse events, including bladder irritation, dysuria, urinary frequency, hematuria, and a flu-like syndrome. While most are manageable, a substantial proportion of patients experience severe adverse events leading to treatment cessation. A recent systematic review highlighted that while general toxicity reporting rates can vary across protocols, severe treatment-limiting adverse events resulting in the complete discontinuation of therapy occurred almost twice as often in patients receiving BCG induction combined with maintenance therapy compared to those undergoing induction alone (154). Rare but life-threatening complications include disseminated BCG infection and BCG-induced sepsis, particularly in immunocompromised individuals.

In the context of intradermal administration or systemic exposure, the Koch phenomenon adds another layer of biological complexity, particularly relevant in the Japanese context. When BCG is administered to individuals with pre-existing *Mycobacterium tuberculosis* infection, an accelerated, localized skin reaction can develop within days. Analysis of 814 reports in Japan revealed that while 71.1% of cases were non-specific reactions, 13.0% represented a true Koch’s phenomenon requiring treatment for latent tuberculosis infection (155). More recent epidemiological data (2013–2019) demonstrated that among 790 infants with Koch-like phenomena and available interferon-gamma release assay (IGRA) results, only 10.3% tested positive, while 89.7% tested negative (156). Importantly, IGRA-positive infants showed a significant correlation with tuberculosis notification rates by prefecture, whereas IGRA-negative infants did not, suggesting that IGRA-positive cases are more likely to represent true infection. No serious reactions due to Koch’s phenomenon were reported in these pediatric cohorts, indicating that while diagnostically significant, this phenomenon is not a major safety concern in itself.

Collectively, these biological, technological, logistical, and safety constraints define the boundaries within which BCG-based platforms must operate. Successful clinical translation of rBCG-based interventions will therefore depend not only on sophisticated genetic engineering but also on standardized surveillance protocols, careful pre-vaccination assessment, and post-vaccination monitoring to ensure safety and maximize therapeutic benefit.

## 8 Conclusion

The proposed paradigm supports two main areas of application. First, BCG, and especially rBCG, can be developed as platform technologies for emergency immunological preparedness. In this context, the controlled induction of trained immunity can provide temporary protection until pathogen-specific countermeasures become available. Second, this same logic can be applied to oncology, where BCG-based interventions can be designed to exploit acute PRR-driven antitumor inflammation while considering the risks defined by the TRIM-Cancer paradox.

Furthermore, BCG-induced pathways can inform therapeutic design, even outside the direct use of BCG itself. Once key convergent signaling nodes are identified, they can be targeted using other pharmacological or biotechnological approaches developed within the mechanistic context established by BCG. Thus, the applicability of this paradigm extends beyond a single vaccine platform to a broader strategy of immunological engineering based on the biology most clearly exposed by BCG.
