# Supplementary material for: The TRIM-cancer paradox: BCG as a programmable vaccine platform and a mechanistic probe for rational immunotherapy design
Source: Front Immunol. 2026 Aug 18;17:1930908. doi: 10.3389/fimmu.2026.1930908 (PMC13532213; doi:10.3389/fimmu.2026.1930908)
Supplement: Supplementary file 1 [file DataSheet1.pdf]

Table 1. The main mechanisms triggered by BCG/TRIM effectors and their impact on immune cells.

Effector	In immunity	In cancer
GM-CSF	APC differentiation and expansion. Monocytes activation: increasing expression of HLA-DR; CD86; TNFα; IL-1β) [1], differentiation to DC, MΦ [2, 3].	Dual role in cancer progression [4, 5]. Increasing: myeloid DCs differentiation, Th1 responses [6], Th9 cell differentiation, antitumor cytotoxic T lymphocyte responses [7]. Excessive - exhaust immune cells, stimulation of cancer growth; insufficient – reduced immune surveillance [5].
Il-1β	Main inflammation and fever activator [8]. Induces autoinflammatory syndromes [9]. Activates NF-κB and MAPK pathways leading to increasing expression of various inflammatory genes [10]. T cell polarization towards Th1 and Th17 subtypes [11]. Activation IFNs signaling and immunity against some viral infections [12].	Resolution of acute inflammation leading to the induction of adaptive anti-tumor responses[13]. Chronic inflammation – risk of cancer, activation tumor growth, metastasis, antiapoptotic effects [11]. In TME: activation of angiogenesis and metastasis [11, 14, 15]. Decreased anti-tumor immune responses [16] activation of MDSC [11]. Immunosuppressive monocytes polarization to IL-10 MΦ, CD8+ T inhibition [17].
IFNs	Induce antiviral, antiproliferative, and immunomodulatory effects; enhance antigen presentation, [18]. Increasing: production of inflammatory cytokines, NK cytotoxic activity, MΦ phagocytosis, DCs maturation and activation, antigen presentation, T cells activation, CD8+T activation and proliferation, CD4+T differentiation to Th1[19-22]. Decreasing expression of anti-inflammatory cytokines [19, 20, 23-25].	Immunosuppression in later stages of tumor progression [26]. Cancer-immunity cycle: antigen release, T cell activation, tumor cell killing [27]. Chronic stimulation – increasing tumor resistance [28]. IFNγ decreases tumor angiogenesis; enhances Treg apoptosis, proinflammatory M1 MΦ antitumor activity immunosuppression, T cell apoptosis, immune tolerance [29]. Cancer cell stemness and metastasis increase [30, 31], EMT [32], increasing PD-L1&IDO dependent immune evasion [33].
TNFα	Major pro-inflammatory cytokine [34, 35]. Activates various immune cells, including MΦs and NFs, increase pathogen phagocytosis; inflammatory mediators increase [34],[36]. Activation of apoptosis [35, 37], ILs production (e.g., IL-1, IL-6); IFNs expression[34, 38]. Excessive activation – chronic inflammation – autoimmune diseases [34].	Pro-inflammatory cancer permissive TME [36, 39]. Immunosuppressive TME [40]. NF-κB activation – increased cancer survival, apoptosis resistance, invasion and metastasis increase [41, 42]. Stimulation of VEGF and, angiogenesis [43].
IL-6	Pro-inflammatory [44]. B cells activation and differentiation; Ig production; humoral immune response [45]. Differentiation of naive CD4+ T to Th17 cells; Treg activity modulation; prevent autoimmunity [46]. C-reactive protein production [47]. Hematopoiesis regulation, HSCs differentiation [48]. Metabolic regulation, insulin sensitivity and lipid metabolism [49].	Dual action in TME - drives malignancy/promotes anti-tumor adaptive immunity [50]. Increased proliferation and survival of cancer cells, activation JAK/STAT3 pathway, cell growth, decrease apoptosis [51]. Stimulation of lymphangiogenesis, VEGF, tumor growth, invasion [52]. Activation of immune escape: increasing of immunosuppressive TME, Tregs and MDSCs amount [53]. Increase N-cadherin, decrease E-cadherin Enhancing of EMT, metastasis, migration, invasion [54].
Th1 polarization	Th1 - immune response against intracellular pathogens, primarily through the production of cytokines like IFNγ and TNFα. Activation MΦ and phagocytosis, increase CD8+T&NK functionality, decreaseTh2 response [55-57].	Anti-tumor immunity: increase production IFNγ, stimulation of cytotoxic activity of CD8+ T/NK cells. MΦ activation, stimulation of differentiation to pro-inflammatory M1 phenotype, phagocytosis, ROS, suppression of tumor growth, angiogenesis and tumor immune evasion [58, 59].
Acetate/ Acetyl-CoA	Acetyl-CoA - H3K27ac acetylation [60] Metabolic supply for Tcells & MΦ, Tregs differentiation, Th2 functionality, anti-inflammatory, inhibiting pro-inflammatory cytokine production, activation of anti-inflammatory M2 MΦ [61-63].	Acetate increases the expressions of SNAI1 and ACSS2 under glucose limitation. ACSS2 knockdown lead to decreased level of SNAI1 and cell migration; ACSS2 overexpression activate SNAI1, histone H3K27 acetylation (H3K27ac), metastasis, migration [60]. ACSS2 catalyzes the conversion of acetate to Ac-CoA [64]. ACSS2 inhibition – impair tumor cell metabolism and potentiate antitumor immunity [64]. Regulation of Ac-CoA metabolic enzymes may support tumor growth and TME survival [65].

Designations:
ACSS2 – Acetyl-CoA synthetase 2;
APC – antigen-presenting cell;
DC – dendritic cell;
EMT – epithelial–mesenchymal transition;
H3K27ac – epigenetic modification to the DNA packaging protein histone H3, acetylation of the lysine residue at N-terminal position 27;
HSCs – hematopoietic stem cells;
JAK/STAT3 - signaling pathway;
MAPK - mitogen-activated protein kinase;
MΦ – macrophages;
M1 MΦ – pro-inflammatory macrophages;
M2 MΦ – anti-inflammatory macrophages;
MDSC – myeloid-derived suppressor cells;
NF-kB – Nuclear Factor Kappa B;
ROS – reactive oxygen species;
SNAI1 – zinc finger protein
Th1, Th9, Th17 – T helper cell 1, 9, 17;
TME – tumor microenvironment;
VEGF – vascular endothelial growth factor;

References:

1. Lotfi N, Zhang GX, Esmaeil N, Rostami A. Evaluation of the effect of GM-CSF blocking on the phenotype and function of human monocytes. Sci Rep. 2020;10(1):1567; doi:10.1038/s41598-020-58131-2
2. Bender AT, Ostenson CL, Giordano D, Beavo JA. Differentiation of human monocytes in vitro with granulocyte-macrophage colony-stimulating factor and macrophage colony-stimulating factor produces distinct changes in cGMP phosphodiesterase expression. Cell Signal. 2004;16(3):365-74; doi:10.1016/j.cellsig.2003.08.009
3. Ushach I, Zlotnik A. Biological role of granulocyte macrophage colony-stimulating factor (GM-CSF) and macrophage colony-stimulating factor (M-CSF) on cells of the myeloid lineage. J Leukoc Biol. 2016;100(3):481-9; doi:10.1189/jlb.3RU0316-144R
4. Alekseenko I, Kuzmich A, Kondratyeva L, Kondratieva S, Pleshkan V, Sverdlov E. Step-by-Step Immune Activation for Suicide Gene Therapy Reinforcement. Int J Mol Sci. 2021;22(17); doi:10.3390/ijms22179376
5. Kumar A, Taghi Khani A, Sanchez Ortiz A, Swaminathan S. GM-CSF: A Double-Edged Sword in Cancer Immunotherapy. Front Immunol. 2022;13:901277; doi:10.3389/fimmu.2022.901277
6. Waller EK. The role of sargramostim (rhGM-CSF) as immunotherapy. Oncologist. 2007;12 Suppl 2:22-6; doi:10.1634/theoncologist.12-S2-22
7. Kim IK, Koh CH, Jeon I, Shin KS, Kang TS, Bae EA, et al. GM-CSF Promotes Antitumor Immunity by Inducing Th9 Cell Responses. Cancer Immunol Res. 2019;7(3):498-509; doi:10.1158/2326-6066.CIR-18-0518
8. Yaseen MM, Abuharfeil NM, Darmani H. The role of IL-1beta during human immunodeficiency virus type 1 infection. Rev Med Virol. 2023;33(1):e2400; doi:10.1002/rmv.2400
9. Broderick L, Hoffman HM. IL-1 and autoinflammatory disease: biology, pathogenesis and therapeutic targeting. Nat Rev Rheumatol. 2022;18(8):448-63; doi:10.1038/s41584-022-00797-1
10. Weber A, Wasiliew P, Kracht M. Interleukin-1 (IL-1) pathway. Sci Signal. 2010;3(105):cm1; doi:10.1126/scisignal.3105cm1
11. Bent R, Moll L, Grabbe S, Bros M. Interleukin-1 Beta-A Friend or Foe in Malignancies? Int J Mol Sci. 2018;19(8); doi:10.3390/ijms19082155
12. Aarreberg LD, Esser-Nobis K, Driscoll C, Shuvarikov A, Roby JA, Gale M, Jr. Interleukin-1beta Induces mtDNA Release to Activate Innate Immune Signaling via cGAS-STING. Mol Cell. 2019;74(4):801-15 e6; doi:10.1016/j.molcel.2019.02.038
13. Mantovani A. Cancer: an infernal triangle. Nature. 2007;448(7153):547-8; doi:10.1038/448547a
14. Rebe C, Ghiringhelli F. Interleukin-1beta and Cancer. Cancers (Basel). 2020;12(7); doi:10.3390/cancers12071791
15. Malkova AM, Gubal AR, Petrova AL, Voronov E, Apte RN, Semenov KN, et al. Pathogenetic role and clinical significance of interleukin-1beta in cancer. Immunology. 2023;168(2):203-16; doi:10.1111/imm.13486
16. Zhou J, Ottewell PD. The role of IL-1B in breast cancer bone metastasis. J Bone Oncol. 2024;46:100608; doi:10.1016/j.jbo.2024.100608
17. Kaplanov I, Carmi Y, Kornetsky R, Shemesh A, Shurin GV, Shurin MR, et al. Blocking IL-1beta reverses the immunosuppression in mouse breast cancer and synergizes with anti-PD-1 for tumor abrogation. Proc Natl Acad Sci U S A. 2019;116(4):1361-9; doi:10.1073/pnas.1812266115
18. Platanias LC. Mechanisms of type-I- and type-II-interferon-mediated signalling. Nat Rev Immunol. 2005;5(5):375-86; doi:10.1038/nri1604
19. Bonjardim CA. Interferons (IFNs) are key cytokines in both innate and adaptive antiviral immune responses--and viruses counteract IFN action. Microbes Infect. 2005;7(3):569-78; doi:10.1016/j.micinf.2005.02.001
20. Mertowska P, Smolak K, Mertowski S, Grywalska E. Immunomodulatory Role of Interferons in Viral and Bacterial Infections. Int J Mol Sci. 2023;24(12); doi:10.3390/ijms241210115

21. Zannikou M, Fish EN, Platanias LC. Signaling by Type I Interferons in Immune Cells: Disease Consequences. Cancers (Basel). 2024;16(8); doi:10.3390/cancers16081600
22. Goldszmid RS, Caspar P, Rivollier A, White S, Dzutsev A, Hieny S, et al. NK cell-derived interferon-gamma orchestrates cellular dynamics and the differentiation of monocytes into dendritic cells at the site of infection. Immunity. 2012;36(6):1047-59; doi:10.1016/j.immuni.2012.03.026
23. Tough DF. Modulation of T-cell function by type I interferon. Immunol Cell Biol. 2012;90(5):492-7; doi:10.1038/icb.2012.7
24. Huber JP, Farrar JD. Regulation of effector and memory T-cell functions by type I interferon. Immunology. 2011;132(4):466-74; doi:10.1111/j.1365-2567.2011.03412.x
25. Kuka M, De Giovanni M, Iannacone M. The role of type I interferons in CD4(+) T cell differentiation. Immunol Lett. 2019;215:19-23; doi:10.1016/j.imlet.2019.01.013
26. Zhou L, Zhang Y, Wang Y, Zhang M, Sun W, Dai T, et al. A Dual Role of Type I Interferons in Antitumor Immunity. Adv Biosyst. 2020;4(11):e1900237; doi:10.1002/adbi.201900237
27. Zhang X, Wang S, Zhu Y, Zhang M, Zhao Y, Yan Z, et al. Double-edged effects of interferons on the regulation of cancer-immunity cycle. Oncoimmunology. 2021;10(1):1929005; doi:10.1080/2162402X.2021.1929005
28. Abdolvahab MH, Darvishi B, Zarei M, Majidzadeh AK, Farahmand L. Interferons: role in cancer therapy. Immunotherapy. 2020;12(11):833-55; doi:10.2217/imt-2019-0217
29. Jorgovanovic D, Song M, Wang L, Zhang Y. Roles of IFN-gamma in tumor progression and regression: a review. Biomark Res. 2020;8:49; doi:10.1186/s40364-020-00228-x
30. Song M, Ping Y, Zhang K, Yang L, Li F, Zhang C, et al. Low-Dose IFNgamma Induces Tumor Cell Stemness in Tumor Microenvironment of Non-Small Cell Lung Cancer. Cancer Res. 2019;79(14):3737-48; doi:10.1158/0008-5472.CAN-19-0596
31. Beziaud L, Young CM, Alonso AM, Norkin M, Minafra AR, Huelsken J. IFNgamma-induced stem-like state of cancer cells as a driver of metastatic progression following immunotherapy. Cell Stem Cell. 2023;30(6):818-31 e6; doi:10.1016/j.stem.2023.05.007
32. Lo UG, Pong RC, Yang D, Gandee L, Hernandez E, Dang A, et al. IFNgamma-Induced IFIT5 Promotes Epithelial-to-Mesenchymal Transition in Prostate Cancer via miRNA Processing. Cancer Res. 2019;79(6):1098-112; doi:10.1158/0008-5472.CAN-18-2207
33. Schalper KA, Carvajal-Hausdorf D, McLaughlin J, Altan M, Velcheti V, Gaule P, et al. Differential Expression and Significance of PD-L1, IDO-1, and B7-H4 in Human Lung Cancer. Clin Cancer Res. 2017;23(2):370-8; doi:10.1158/1078-0432.CCR-16-0150
34. Jang DI, Lee AH, Shin HY, Song HR, Park JH, Kang TB, et al. The Role of Tumor Necrosis Factor Alpha (TNF-alpha) in Autoimmune Disease and Current TNF-alpha Inhibitors in Therapeutics. Int J Mol Sci. 2021;22(5); doi:10.3390/ijms22052719
35. van Loo G, Bertrand MJM. Death by TNF: a road to inflammation. Nat Rev Immunol. 2023;23(5):289-303; doi:10.1038/s41577-022-00792-3
36. Laha D, Grant R, Mishra P, Nilubol N. The Role of Tumor Necrosis Factor in Manipulating the Immunological Response of Tumor Microenvironment. Front Immunol. 2021;12:656908; doi:10.3389/fimmu.2021.656908
37. van Deventer SJ. Transmembrane TNF-alpha, induction of apoptosis, and the efficacy of TNF-targeting therapies in Crohn's disease. Gastroenterology. 2001;121(5):1242-6; doi:10.1053/gast.2001.29035
38. Faraj SS, Jalal PJ. IL1beta, IL-6, and TNF-alpha cytokines cooperate to modulate a complicated medical condition among COVID-19 patients: case-control study. Ann Med Surg (Lond). 2023;85(6):2291-7; doi:10.1097/MS9.0000000000000679
39. Ham B, Fernandez MC, D'Costa Z, Brodt P. The diverse roles of the TNF axis in cancer progression and metastasis. Trends Cancer Res. 2016;11(1):1-27;
40. Alim LF, Keane C, Souza-Fonseca-Guimaraes F. Molecular mechanisms of tumour necrosis factor signalling via TNF receptor 1 and TNF receptor 2 in the tumour microenvironment. Curr Opin Immunol. 2024;86:102409; doi:10.1016/j.coi.2023.102409
41. Wu Y, Zhou BP. TNF-alpha/NF-kappaB/Snail pathway in cancer cell migration and invasion. Br J Cancer. 2010;102(4):639-44; doi:10.1038/sj.bjc.6605530
42. Tang D, Tao D, Fang Y, Deng C, Xu Q, Zhou J. TNF-Alpha Promotes Invasion and Metastasis via NF-Kappa B Pathway in Oral Squamous Cell Carcinoma. Med Sci Monit Basic Res. 2017;23:141-9; doi:10.12659/msmbr.903910
43. Shin MR, Kang SK, Kim YS, Lee SY, Hong SC, Kim EC. TNF-alpha and LPS activate angiogenesis via VEGF and SIRT1 signalling in human dental pulp cells. Int Endod J. 2015;48(7):705-16; doi:10.1111/iej.12396
44. Tanaka T, Narazaki M, Kishimoto T. IL-6 in inflammation, immunity, and disease. Cold Spring Harb Perspect Biol. 2014;6(10):a016295; doi:10.1101/cshperspect.a016295
45. Dienz O, Eaton SM, Bond JP, Neveu W, Moquin D, Noubade R, et al. The induction of antibody production by IL-6 is indirectly mediated by IL-21 produced by CD4+ T cells. J Exp Med. 2009;206(1):69-78; doi:10.1084/jem.20081571
46. Li B, Jones LL, Geiger TL. IL-6 Promotes T Cell Proliferation and Expansion under Inflammatory Conditions in Association with Low-Level RORgammat Expression. J Immunol. 2018;201(10):2934-46; doi:10.4049/jimmunol.1800016
47. Ngwa DN, Pathak A, Agrawal A. IL-6 regulates induction of C-reactive protein gene expression by activating STAT3 isoforms. Mol Immunol. 2022;146:50-6; doi:10.1016/j.molimm.2022.04.003
48. Tie R, Li H, Cai S, Liang Z, Shan W, Wang B, et al. Interleukin-6 signaling regulates hematopoietic stem cell emergence. Exp Mol Med. 2019;51(10):1-12; doi:10.1038/s12276-019-0320-5
49. Li YS, Ren HC, Cao JH. Roles of Interleukin-6-mediated immunometabolic reprogramming in COVID-19 and other viral infection-associated diseases. Int Immunopharmacol. 2022;110:109005; doi:10.1016/j.intimp.2022.109005

50. Fisher DT, Appenheimer MM, Evans SS. The two faces of IL-6 in the tumor microenvironment. Semin Immunol. 2014;26(1):38-47; doi:10.1016/j.smim.2014.01.008
51. Raskova M, Lacina L, Kejik Z, Venhauerova A, Skalickova M, Kolar M, et al. The Role of IL-6 in Cancer Cell Invasiveness and Metastasis-Overview and Therapeutic Opportunities. Cells. 2022;11(22); doi:10.3390/cells11223698
52. Zhao G, Zhu G, Huang Y, Zheng W, Hua J, Yang S, et al. IL-6 mediates the signal pathway of JAK-STAT3-VEGF-C promoting growth, invasion and lymphangiogenesis in gastric cancer. Oncol Rep. 2016;35(3):1787-95; doi:10.3892/or.2016.4544
53. Bent EH, Millan-Barea LR, Zhuang I, Goulet DR, Frose J, Hemann MT. Microenvironmental IL-6 inhibits anti-cancer immune responses generated by cytotoxic chemotherapy. Nat Commun. 2021;12(1):6218; doi:10.1038/s41467-021-26407-4
54. Zhang KW, Wang D, Cai H, Cao MQ, Zhang YY, Zhuang PY, et al. IL-6 plays a crucial role in epithelial-mesenchymal transition and pro-metastasis induced by sorafenib in liver cancer. Oncol Rep. 2021;45(3):1105-17; doi:10.3892/or.2021.7926
55. Ekkens MJ, Shedlock DJ, Jung E, Troy A, Pearce EL, Shen H, et al. Th1 and Th2 cells help CD8 T-cell responses. Infect Immun. 2007;75(5):2291-6; doi:10.1128/IAI.01328-06
56. Muraille E, Leo O, Moser M. TH1/TH2 paradigm extended: macrophage polarization as an unappreciated pathogen-driven escape mechanism? Front Immunol. 2014;5:603; doi:10.3389/fimmu.2014.00603
57. Howard FHN, Kwan A, Winder N, Mughal A, Collado-Rojas C, Muthana M. Understanding Immune Responses to Viruses-Do Underlying Th1/Th2 Cell Biases Predict Outcome? Viruses. 2022;14(7); doi:10.3390/v14071493
58. Nonaka K, Saio M, Umemura N, Kikuchi A, Takahashi T, Osada S, et al. Th1 polarization in the tumor microenvironment upregulates the myeloid-derived suppressor-like function of macrophages. Cell Immunol. 2021;369:104437; doi:10.1016/j.cellimm.2021.104437
59. Lee J, Lozano-Ruiz B, Yang FM, Fan DD, Shen L, Gonzalez-Navajas JM. The Multifaceted Role of Th1, Th9, and Th17 Cells in Immune Checkpoint Inhibition Therapy. Front Immunol. 2021;12:625667; doi:10.3389/fimmu.2021.625667
60. Yao L, Jiang L, Zhang F, Li M, Yang B, Zhang F, et al. Acetate promotes SNAI1 expression by ACSS2-mediated histone acetylation under glucose limitation in renal cell carcinoma cell. Biosci Rep. 2020;40(6); doi:10.1042/BSR20200382
61. Qiu J, Villa M, Sanin DE, Buck MD, O'Sullivan D, Ching R, et al. Acetate Promotes T Cell Effector Function during Glucose Restriction. Cell Rep. 2019;27(7):2063-74 e5; doi:10.1016/j.celrep.2019.04.022
62. Zhang S, Lv K, Liu Z, Zhao R, Li F. Fatty acid metabolism of immune cells: a new target of tumour immunotherapy. Cell Death Discov. 2024;10(1):39; doi:10.1038/s41420-024-01807-9
63. Xu M, Jiang Z, Wang C, Li N, Bo L, Zha Y, et al. Acetate attenuates inflammasome activation through GPR43-mediated Ca(2+)-dependent NLRP3 ubiquitination. Exp Mol Med. 2019;51(7):1-13; doi:10.1038/s12276-019-0276-5
64. Miller KD, O'Connor S, Pniewski KA, Kannan T, Acosta R, Mirji G, et al. Acetate acts as a metabolic immunomodulator by bolstering T-cell effector function and potentiating antitumor immunity in breast cancer. Nat Cancer. 2023;4(10):1491-507; doi:10.1038/s43018-023-00636-6
65. Guertin DA, Wellen KE. Acetyl-CoA metabolism in cancer. Nat Rev Cancer. 2023;23(3):156-72; doi:10.1038/s41568-022-00543-5
