# Supplementary material for: The TRIM-cancer paradox: BCG as a programmable vaccine platform and a mechanistic probe for rational immunotherapy design
Source: Front Immunol. 2026 Aug 18;17:1930908. doi: 10.3389/fimmu.2026.1930908 (PMC13532213; doi:10.3389/fimmu.2026.1930908)
Supplement: Supplementary file 2 [file DataSheet2.zip › Supplementary Table S2.html]

TRIM–Cancer Paradox: Therapeutic Target Matrix


# TRIM–Cancer Paradox: Therapeutic Target and Agent Matrix

Each row represents one molecular target or pharmacological agent. Left
column: effect on BCG-induced trained immunity (TRIM). Right column:
effect on cancer or other pathological condition. Arrows indicate
direction: ↑ promotes, ↓ suppresses, ± ambiguous/bidirectional. Where the
role in cancer is unknown or controversial, the cell is marked
**?** with commentary. Sections are separated by colored
subheadings. Note that this table provides a general principle for BCG
modification that may potentially enhance its anticancer properties, and
it should not be regarded as a concrete or experimentally proven strategy.

PRR /
Transcription
mTOR / Glycolysis
/ Warburg
Metabolite–epigenetic axis
Epigenetic
modifiers
TRIM ↑
TRIM ↓
TRIM ± dual
TRIM ?
unknown

⬇ Download CSV

| Target / Agent | Effect on TRIM | TRIM direction | Effect on pathological condition | Condition | A possible solution for the anti-cancer modification of BCG |
| --- | --- | --- | --- | --- | --- |
| SECTION I — PRR / Transcription factor signaling | | | | | |
| **TLR2/TLR4 agonists (e.g., MPLA, BCG cell-wall components)** | Acute NF-κB activation → IL-6, IL-1β, TNFα production; initiation of TRIM in monocytes/macrophages | TRIM ↑ | Acute: antitumor innate activation (bladder, melanoma). Chronic: NF-κB-dependent survival signaling, MDSC recruitment, angiogenesis, metastasis | Cancer (dual) | BCG cell wall |
| **NOD2 agonist (muramyl dipeptide, MDP)** | Obligatory for BCG-induced TRIM; NOD2-KO abrogates TRIM induction in monocytes | TRIM ↑ | NOD2 loss → protumorigenic M2-like TAM phenotype, accelerated lung adenocarcinoma progression. NOD2 agonism may restore M1-like balance | Cancer (dual) | BCG cell wall |
| **Dectin-1 agonist (β-glucan, e.g., laminarin, zymosan)** | Dectin-1 → AKT/mTOR → glycolytic shift → acetyl-CoA/succinate → epigenetic TRIM imprinting | TRIM ↑ | Trained immunity inducers from β-glucan suppress melanoma (ABB i16) and potentiate ICI therapy; anti-tumor via myeloid reprogramming | Cancer (anti) | BCG cell wall |
| **NF-κB inhibitor (e.g., bortezomib, PDTC, Bay 11-7082)** | Blocks TRIM induction by suppressing acute inflammatory gene transcription; impairs IL-1β, TNFα, IL-6 production downstream of BCG | TRIM ↓ | Anti-cancer: inhibits tumor cell survival, angiogenesis, metastasis; NF-κB blockade restores immune surveillance in many tumor types | Cancer (anti) | NF-κB blockade factors expressed by BCG: IκBα, A20/TNFAIP3, CYLD |
| SECTION II — mTOR / Glycolysis / Warburg axis | | | | | |
| **Rapamycin (sirolimus) / mTOR inhibitors (rapalogs: everolimus, temsirolimus)** | Inhibits AKT/mTOR → blocks glycolytic reprogramming → suppresses TRIM induction; rapamycin abolishes trained phenotype in β-glucan- and CpG-trained monocytes | TRIM ↓ | Anti-cancer: mTOR inhibition reduces proliferation, survival, PD-L1 expression; approved for RCC, breast cancer, mantle cell lymphoma; also promotes autophagy → can paradoxically increase drug tolerance in some contexts | Cancer (anti, context-dep.) | DEPTOR (or other mTOR1/2 inhibitor protein) encoded by BCG + small molecules inhibiting mTOR pathway |
| **Metformin (AMPK activator / Complex I inhibitor)** | AMPK activation inhibits mTOR → suppresses glycolysis-dependent TRIM reprogramming; reduces TRIM magnitude in monocytes in vitro | TRIM ↓ | Anti-diabetic (T2DM, first-line). Anti-cancer: inhibits Warburg glycolysis, OXPHOS; reduces proliferation, PD-L1 expression; reprograms TME toward immune-permissive state; enhances T cell memory | Cancer (anti) / Diabetes (therapeutic) | Upstream activators of АМРК - LKB1, TAK1 etc., encoded by BCG + small molecules AMPK activators |
| **2-Deoxyglucose (2-DG)** | Competitive glycolysis inhibitor → reduces acetyl-CoA and immunometabolite flux → suppresses TRIM induction in monocytes and macrophages | TRIM ↓ | Anti-cancer: synergizes with metformin to inhibit proliferation, migration, angiogenesis in ovarian, breast, glioma models; anti-diabetic potential (reduces glucose-driven metabolic dysregulation) | Cancer (anti) / Diabetes (investigational) | Encoded by BCG upsthream pathways to reduce acetyl-CoA: INSIG → SREBP1 → ACLY → acetyl-CoA↓ |
| **HIF-1α inhibitor (e.g., PX-478, PT2977/belzutifan)** | HIF-1α downstream of mTOR is required for glycolytic amplification and IL-1β-driven TRIM; HIF-1α inhibition blunts TRIM induction | TRIM ↓ | Anti-cancer: HIF-1α drives Warburg effect, VEGF, immune evasion; dual HIF-1/2 inhibitors under clinical evaluation in combination with ICI | Cancer (anti) | Encoded by BCG HIF-1α inhibitors: PHD1/2/3, VHL, FIH etc. |
| SECTION III — Metabolite–epigenetic axis | | | | | |
| **Dimethyl fumarate (DMF) / Monomethyl fumarate (MMF)** | Fumarate accumulation → KDM5 inhibition → H3K4me3 enrichment at IL-6/TNF promoters → TRIM epigenetic imprinting; DMF activates Nrf2, upregulates ULBP2/5 on stressed cells → NK cell recognition | TRIM ↑ (partial) | Anti-cancer: enhances NK cell cytotoxicity via ULBP ligand upregulation; colitis-associated cancer: low-dose protective, high-dose pro-oxidant (context-dependent); autoimmune disease (MS): approved; tumor cell killing via ROS/ferroptosis in some models | Cancer (dual, dose-dep.) / Autoimmune | Encoded by BCG shedding resistant ULPBs and/or ADAM inhibitors (TIMP) |
| **Succinate / SUCNR1 pathway (SDH inhibitor: itaconate, malonate)** | Succinate stabilizes HIF-1α → IL-1β → acute TRIM induction; itaconate (via IRG1/ACOD1) inhibits SDH → dampens succinate oxidation → anti-inflammatory feedback; B-glucan TRIM requires suppression of ACOD1 | TRIM ↑ (succinate) / TRIM ↓ (itaconate) | Cancer: tumor-derived succinate → SUCNR1 on TAMs → PI3K-HIF-1α → immunosuppression; cancer cell-intrinsic itaconate → TFEB-mediated antigen presentation → improved T cell immunity and ICI response; SDH-mutant tumors accumulate succinate (oncometabolite, paraganglioma, GIST) | Cancer (dual) | BCG strain expressing IRG1/ACOD1 to increase itaconate and blunt succinate-SUCNR1-HIF1α signaling in TAMs |
| **Mevalonate pathway inhibitors (statins: atorvastatin, simvastatin)** | Mevalonate → IGF1R/mTOR → TRIM imprinting (required for β-glucan-induced training); statins block HMGCR → deplete mevalonate → reduce TRIM magnitude in monocytes | TRIM ↓ | Anti-cancer: statins inhibit protein prenylation (RAS/Rho), reduce metastasis, improve survival in epidemiological studies; cancer stem cell programs overlap with TRIM metabolic signatures; mevalonate immune surveillance via NKG2D ligands on stressed tumor cells | Cancer (anti) | Encoded by BCG - mutant SREBP (activation impaired), SCAP (SREBP Cleavage-Activating Protein), Insig-1/2 blocking HMGCR and other mavalonate pathway blokers |
| **α-KG supplement / IDH-mutant inhibitors (enasidenib, ivosidenib)** | High αKG/succinate ratio → JMJD3-dependent H3K27me3 demethylation → context-dependent M2-like macrophage polarization; αKG supplementation skews TAMs toward M2 in TME | TRIM — (ambiguous) | Cancer: IDH1/2 mutations produce 2-HG (competitive antagonist of αKG-dependent dioxygenases) → epigenetic dysregulation → glioma, AML; IDH inhibitors (enasidenib, ivosidenib) reverse 2-HG-mediated silencing; OXGR1/αKG signaling in TAMs suppresses MHC-II → immune evasion | Cancer (anti via IDH-i; pro via OXGR1) | Encoded by BCG - mutant IDH (mIDH1/2) |
| **Acetyl-CoA / ACLY inhibitor (e.g., BMS-303141, hydroxycitrate)** | BCG-driven glycolysis → ↑ acetyl-CoA → HATs → H3K27ac at inflammatory promoters → TRIM; ACLY inhibition reduces acetyl-CoA pool → dampens TRIM-associated histone acetylation | TRIM ↓ | Anti-cancer: ACLY overexpression supports super-enhancer-driven oncogene expression; ACLY inhibitors reduce tumor cell growth in vitro and in vivo; bempedoic acid (cholesterol-lowering ACLY inhibitor): FDA-approved for cardiovascular risk reduction | Cancer (anti, experimental) | Encoded by BCG upsthream pathways to reduce acetyl-CoA: INSIG → SREBP1 → ACLY → acetyl-CoA↓ |
| **NAD+ precursors (NMN, NR, nicotinamide)** | Restore NAD+ → support SIRT1/SIRT3 activity → deacetylation of metabolic regulators → can preserve or modulate TRIM depending on context; butyrate-mediated HDAC3 inhibition antagonizes NAD+-dependent TRIM | TRIM ± (NAD+ restoration) | Cancer: NAD+ depletion in TME supports immunosuppression and T cell exhaustion; NMN/NR restore NK and T cell function in aged/immunosuppressed hosts; excess NAD+ in cancer cells fuels PARP-mediated survival → PARP inhibitors (olaparib) cut this supply | Cancer (context-dep.) / Aging / Metabolic syndrome | Caution: NAD+ aids in the repair of DNA in cancer cells but is necessary to fuel immune cells |
| SECTION IV — Epigenetic modifiers (writers / erasers) | | | | | |
| **G9a/EHMT2 inhibitor (BIX-01294, UNC0638)** | G9a deposits repressive H3K9me2 at inflammatory loci; G9a expression decreases during TRIM induction; pharmacological G9a inhibition amplifies TRIM responses and synergizes with BCG in bladder cancer models | TRIM ↑ | Cancer: G9a overexpression silences tumor suppressor genes, supports EMT; G9a inhibitors restore tumor suppressor expression and enhance immunogenicity; in breast cancer, G9a disrupts iron homeostasis to promote growth | Cancer (anti) | miR-217, miR-142-3p supress G9a/EHMT2. Potential dual role, depending on cancer type |
| **EZH2 inhibitor (tazemetostat / valemetostat)** | EZH2 deposits H3K27me3 → represses inflammatory loci; EZH2 inhibition may derepress pro-inflammatory programs and increase TRIM permissiveness, but direct TRIM induction data in myeloid cells are limited | TRIM ? (possibly ↑, limited data) | Anti-cancer: EZH2 inhibits TSG expression and immune gene programs; tazemetostat FDA-approved (follicular lymphoma, epithelioid sarcoma); transient EZH2 inhibition preserves T cell stemness (TCF1) and enhances CAR-T efficacy; also under evaluation in combination with anti-PD-1 | Cancer (anti) | Encoded by BCG - p21 (CDKN1A) expression as universal anti-cancer stratege |
| **HDAC inhibitors (vorinostat/SAHA, romidepsin, panobinostat, butyrate)** | Butyrate/HDAC3 inhibition blocks H3K27ac/H3K9ac accumulation at inflammatory promoters → strongly suppresses TRIM induction; vorinostat similarly impairs trained phenotype via pan-HDAC inhibition | TRIM ↓ | Anti-cancer: HDAC-i restore TSG expression (histone re-acetylation in cancer); vorinostat FDA-approved (CTCL); panobinostat approved (multiple myeloma); HDAC-i + ICI combinations in multiple trials; TGF-β-mediated HDAC upregulation in chronic viral infection (restored by HDAC-i) | Cancer (anti) | Encoded by BCG – CaMK and SIK kinases to phosphorylate class IIa HDACs, relieving repression of pro‑apoptotic and differentiation programs |
| **BET/BRD4 inhibitor (JQ1, I-BET762, ABBV-075)** | JQ1 dampens H3K27ac-dependent super-enhancer-driven transcription; impairs GM-CSF-driven macrophage self-renewal and IL-4-induced M2 polarization → may suppress TRIM-related pro-inflammatory programs in some contexts | TRIM ↓ (partial) | Anti-cancer: JQ1 downregulates PD-L1 (BRD4-dependent), increases MHC-I, reduces Treg infiltration, synergizes with anti-PD-1 in CRC, NSCLC, HCC; ABBV-075 depletes pro-tumor CCR2+ TAMs in ovarian cancer | Cancer (anti) | BET inhibitor NHWD-870 in combination with BCG vaccine enhances antitumor efficacy. Search for BCG-encoded BET inhibitors |
| **KDM5 inhibitor (e.g., CPI-455, KDM5-C70)** | Fumarate-mediated KDM5 inhibition in TRIM → H3K4me3 enrichment at IL-6/TNF promoters → epigenetic memory; direct KDM5 inhibitors may recapitulate fumarate-mediated TRIM enhancement | TRIM ↑ (potential) | Cancer: KDM5A/B demethylate H3K4me3 at TSG promoters → epigenetic silencing in lung, breast, leukemia; KDM5 inhibitors restore H3K4me3 at TSG promoters and sensitize to ICI; anti-tumor activity preclinically | Cancer (anti, experimental) | miR-137 supress KDM5 |
| **miR-137 supress KDM5** | αKG-dependent JMJD3 removes H3K27me3 from inflammatory loci during M1-like and TRIM activation; JMJD3 inhibition blocks derepression of these genes and dampens TRIM | TRIM ↓ | Cancer: JMJD3 is context-dependent oncogene/TSG; KDM6B promotes proliferation in multiple myeloma and prostate cancer (KDM6B inhibition → anti-tumor); JMJD3 supports M2 TAM polarization in TME → JMJD3-i may restore M1 balance | Cancer (dual, context-dep.) | miR-146a supress JMJD3. Context dependent role |

**Legend:**
TRIM ↑ = agent promotes trained immunity induction or maintenance. TRIM ↓
= agent suppresses trained immunity. TRIM ± = context-dependent or
dose-dependent bidirectional effect. TRIM ? = role in trained immunity not
established; labelled "possibly anti or pro cancer" where cancer role is
also uncertain. Condition column: Cancer (anti) = established or strongly
supported anti-cancer evidence; Cancer (dual) = acute activation
anti-tumor, chronic activation pro-tumor; Cancer (context-dep.) = depends
on dose, timing, or tumor type; Diabetes (therapeutic) = approved or
primary use in diabetes/metabolic disease. miR - microRNA (miRNA) is a
small RNA molecule that inhibits the expression of a specific target gene.
Currently, there are no studies demonstrating the use of BCG as a vector
for microRNA precursor expression. However, BCG is known to modulate
cellular microRNA levels. In the context of the table and the discussed
recombinant BCG constructs, this suggests that employing BCG in this
setting is unlikely, unless one resorts to complex and unconventional
engineering solutions.
